# Nitroxoline exerts potent anti-*Aspergillus fumigatus* activity by disrupting copper homeostasis and inducing oxidative stress

**DOI:** 10.1128/aac.01831-25

**Published:** 2026-03-31

**Authors:** Yiru Zhang, Yuxin Han, Dan Cao, Xin Yuan, Pusheng Xu, Yanghui Xiang, Yi Li, Ying Zhang

**Affiliations:** 1 State Key Laboratory for Diagnosis and Treatment of Infectious Diseases, National Clinical Research Center for Infectious Diseases, China-Singapore Belt and Road Joint Laboratory on Infection Research and Drug Development, National Medical Center for Infectious Diseases, Collaborative Innovation Center for Diagnosis and Treatment of Infectious Diseases, The First Affiliated Hospital, Zhejiang University School of Medicine 26441, Hangzhou, Zhejiang, China; 2 Yuhang Institute for Collaborative Innovation and Translational Research in Life Sciences and Technology, Hangzhou, Zhejiang, China; 3 Jinan Microecological Biomedicine Shandong Laboratory, Jinan, China; University of Iowa, Iowa City, Iowa, USA

**Keywords:** *Aspergillus fumigatus*, nitroxoline, antifungal activity, copper homeostasis, ROS

## Abstract

Invasive pulmonary aspergillosis (IPA) caused by *Aspergillus fumigatus* remains a clinical challenge due to limited therapies and rising antifungal resistance. Here, we evaluated nitroxoline (NTX), an FDA-approved 8-hydroxyquinoline derivative, for its antifungal potential and mechanism of action. NTX exhibited potent *in vitro* activity against 64 clinical isolates and the AF293 reference strain, delaying conidial maturation and significantly improving survival, reducing pulmonary fungal burden, and alleviating lung inflammation and hyphal invasion in an IPA mouse model. Transcriptomic analysis revealed marked downregulation of the high-affinity Cu transporter *ctrC* and superoxide dismutase *sodB*, disrupting the copper homeostasis-oxidative stress axis. Copper supplementation restored hyphal growth and intracellular ROS levels, supporting a mechanism of copper deprivation-induced oxidative stress. These findings demonstrate that NTX exerts strong anti-*A*. *fumigatus* effects *in vitro* and *in vivo* by perturbing copper homeostasis and inducing oxidative stress, highlighting its unique antifungal potential and providing a conceptual basis for metal-targeted therapeutic strategies against IPA and drug-resistant fungal infections.

## INTRODUCTION

Invasive pulmonary aspergillosis (IPA), primarily caused by the mold *A. fumigatus*, is a lethal opportunistic fungal infection afflicting immunocompromised patients, including those with hematologic malignancies, organ transplant recipients, and critically ill individuals (1). Despite advancements in antifungal therapies, the mortality rate for IPA remains unacceptably high, often exceeding 50%, due to the inherent resistance of the fungus and the toxicity of available treatments, such as nephrotoxicity associated with Amphotericin B (2–5). Consequently, there is an urgent and unmet need to develop novel, safe, cost-effective, and mechanistically distinct antifungal strategies.

In the context of protracted drug development cycles and soaring costs, drug repurposing has emerged as an efficient and cost-effective avenue for discovering new anti-infective agents, given their established pharmacokinetic and safety profiles (6). Nitroxoline (NTX), an 8-hydroxy-5-nitroquinoline derivative, is an orally administered antimicrobial with over 50 years of clinical history, primarily used for treating urinary tract infections (7, 8). Leveraging its favorable safety profile, well-defined toxicity spectrum, and low cost, NTX has recently been shown to have activity against several pathogens, including those causing tuberculosis and candidiasis (9, 10). Although some evidence suggests that NTX may also inhibit *Aspergillus* species, its activity against *A. fumigatus*, particularly its mode of action, has not been systematically characterized (11).

From a molecular perspective, NTX belongs to the class of 8-hydroxyquinoline (8-HQ) compounds, whose broad-spectrum bioactivity is closely linked to their ability to chelate metal ions and act as ionophores (12, 13). These compounds facilitate the transmembrane transport of transition metal ions, such as Cu²^+^ and Zn²^+^, disrupting intracellular metal homeostasis (14–16). For pathogenic fungi, especially *A. fumigatus*, survival, virulence expression, and resistance to host immune pressure (such as macrophage-induced metal starvation or metal-overload stress) are heavily reliant on the precise regulation of metal ion uptake and efflux, particularly copper and iron (17, 18). Therefore, targeting fungal metal homeostasis, particularly copper homeostasis, is recognized as a promising strategy for the development of novel antifungal agents (19, 20). While NTX was found to have activity against *C. albicans*, its antifungal activity and mechanistic basis in *A. fumigatus* remain insufficiently understood, necessitating further investigation into whether and how NTX disrupts copper homeostasis and redox balance to mediate this antifungal activity (10).

To address this knowledge gap, this study evaluated the *in vitro* and *in vivo* activity of NTX against *A. fumigatus* and explored its potential mechanisms of action through transcriptomic profiling. Our results show that NTX significantly affects the expression of genes involved in metal homeostasis and oxidative stress responses. Building on these findings, we further validated that NTX likely exerts its antifungal effects by disrupting copper ion homeostasis and inducing oxidative stress. This study not only provides important preclinical evidence for the repurposing of NTX as a therapeutic option for *A. fumigatus* infections but also offers new insights and theoretical support for developing antifungal strategies targeting metal homeostasis.

## MATERIALS AND METHODS

### Fungal strains and reagents

*A. fumigatus* AF293 was obtained from Mingzhou Biotechnology (Ningbo, China), and 64 clinical isolates were provided by the Clinical Laboratory of the First Affiliated Hospital of Zhejiang University. All strains were cultured on PDA at 37°C for 3–5 days. Conidia were harvested with PBS containing 0.1% Tween-80, filtered through a 40 µm strainer, counted with a hemocytometer, and adjusted to required concentrations. For *in vitro* assays, NTX, VRC, and AmB were dissolved in DMSO, sterilized by 0.22 µm filtration, aliquoted, and stored at −20°C. For *in vivo* use, NTX and VRC were dissolved in 10% sulfobutyl ether-β-cyclodextrin (SBE-β-CD) via ultrasonication and sterilized by filtration. Cyclophosphamide (CTX) and CuSO₄ were freshly prepared in sterile ddH_2_O, filtered, and used immediately.

### Determination of MIC

MICs of VRC, AmB, and NTX against AF293 and 64 clinical isolates were determined following EUCAST E.DEF 9.3.2 using broth microdilution. Briefly, assays were performed in double-strength RPMI-1640 supplemented with 2% glucose (pH 7.0). Conidial suspensions were adjusted to 1–2.5 × 10^5^ CFU/mL. Drugs were serially diluted to final concentrations of 0.0125–16 µg/mL, and plates were incubated at 37°C for 48 h. MICs were visually read as the lowest concentration showing complete inhibition of fungal growth.

### Morphological observation

AF293 conidia (1 × 10^6^ conidia/mL) were incubated in potato dextrose broth (PDB) with VRC or NTX at 1× or 2× MIC, with a drug-free control. Suspensions were added to 24-well plates containing sterile coverslips and incubated statically at 37°C. After 6 and 14 h, coverslips were mounted on slides, and conidial germination and hyphal morphology were observed using an Olympus IX73 inverted microscope. For scanning electron microscopy (SEM), AF293 conidia (1 × 10^6^ conidia/mL) were cultured in PDB with VRC or NTX at 1 × MIC or without drug. After 6 and 14 h at 37°C with shaking, cells were collected, washed, fixed in 2.5% glutaraldehyde at 4°C overnight, post-fixed in osmium tetroxide, dehydrated through graded ethanol, critical-point dried, sputter-coated with gold, and imaged by SEM.

### Animal model and *in vivo* therapeutic efficacy assessment

**Fig 1:**
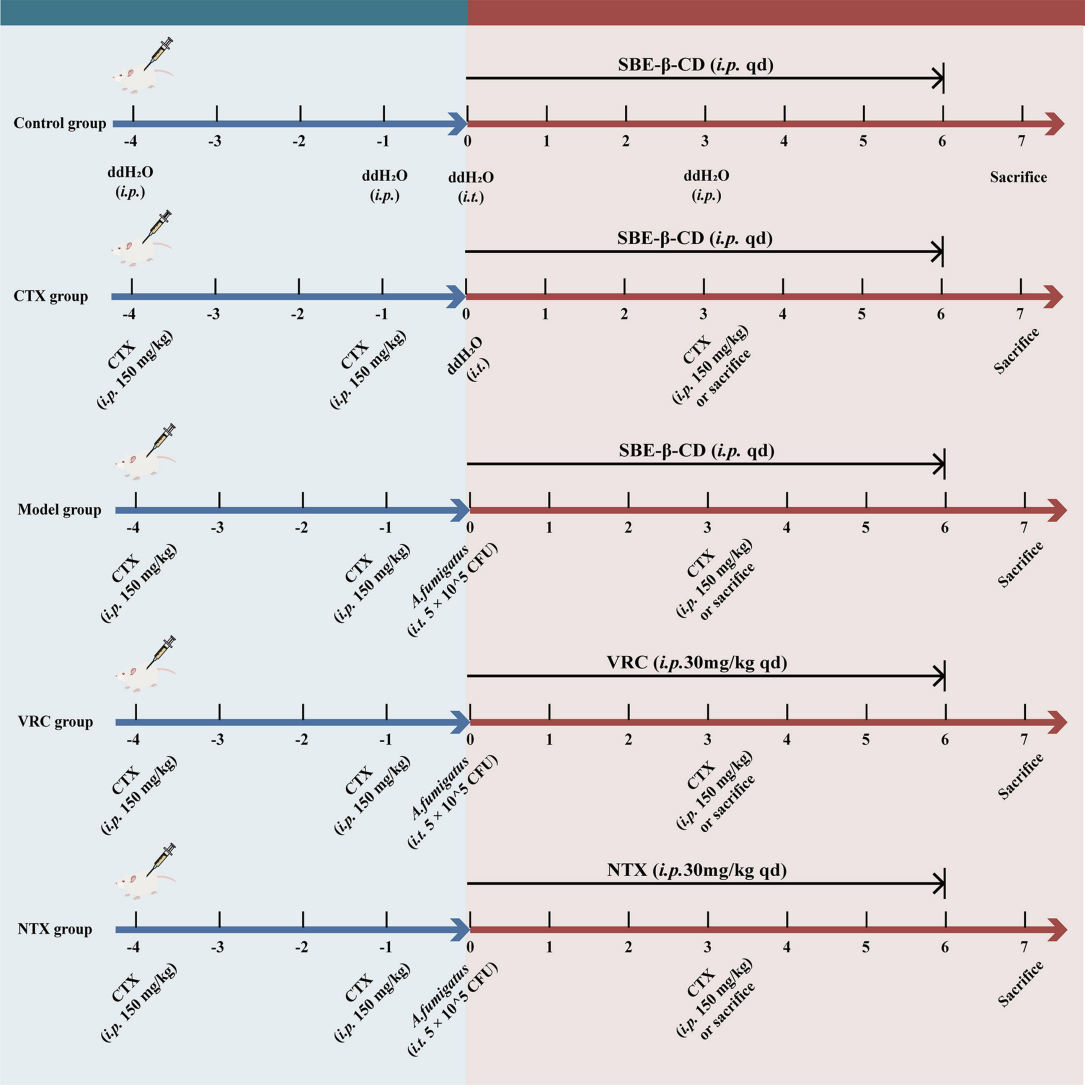
Experimental procedures for mice in different experimental groups*.* SBE-β-CD, sulfobutylether-β-cyclodextrin; CTX, cyclophosphamide; VRC, voriconazole; NTX, nitroxoline.

Female BALB/c mice (6–8 weeks, 18–22 g) were obtained from the Laboratory Animal Research Center of Hangzhou Medical College and housed under SPF conditions. All procedures were approved by the Institutional Animal Care and Use Committee of the First Affiliated Hospital, Zhejiang University. Mice were euthanized by cervical dislocation when body weight loss exceeded 25% or irreversible dyspnea occurred. Immunosuppression was induced by intraperitoneal CTX (150 mg/kg) on days −4, −1, and +3 (Fig. 1). WBC counts from orbital plexus blood confirmed immunosuppression. On day 0, the Model, VRC, and NTX groups were intranasally inoculated with 50 µL AF293 conidia (1 × 10^7^ conidia/mL), while the Control and CTX groups received ddH_2_O. Two hours later, VRC and NTX groups received intraperitoneal injections of voriconazole or nitroxoline (30 mg/kg/day) for 6 days, and others received vehicle. The intraperitoneal route and dose were selected based on prior studies in non-urinary disease models to evaluate systemic efficacy and to align with the common dose of voriconazole used in IPA models for initial functional comparison (21, 22). For survival, 20 mice per group (CTX, Model, VRC, NTX) were monitored for 14 days. For fungal burden and histopathology, five groups (Control, CTX, Model, VRC, NTX) with at least 10 mice per group were used. Lungs were collected on day 3 for CFU enumeration on PDA plates and on days 3 and 7 for histological analysis. The tissues were fixed and stained with hematoxylin and eosin (H&E) and periodic acid-Schiff methenamine (PASM) to evaluate inflammation and hyphal invasion, respectively.

### Transcriptomic and bioinformatic analyses

AF293 conidia (1 × 10^7^) were inoculated into 10 mL PDB and treated with nitroxoline (NTX, 2 µg/mL) or DMSO (control), with three biological replicates per group. Cultures were incubated at 37°C, 220 rpm for 4 h, washed with PBS, and collected for RNA-seq. Samples were flash-frozen and sent to Majorbio (Shanghai) for transcriptome analysis. RNA passing QC was used for cDNA library construction and sequenced on an Illumina NovaSeq X Plus platform. Clean reads were mapped to the AF293 genome (GCF_000002655.1), and DEGs were identified using DESeq2 (FDR < 0.05, |log_2_FC| ≥ 1). GO, KEGG, and GSEA analyses were performed using Goatools, KOBAS, and GSEA software. Peroxisome-related DEGs were analyzed in STRING (confidence ≥ 0.4) and Cytoscape (v3.10.4). Homologous CtrC and SodB sequences were retrieved from NCBI and UniProt. Phylogeny was built in MEGA 12.0 using the Neighbor-Joining method with 1,000 bootstrap replicates. Protein structures were modeled using SWISS-MODEL or obtained from the PDB, and NTX docking was performed with AutoDock Vina and visualized in PyMOL. The transcriptome raw sequencing data generated in this study have been deposited in the National Center for Biotechnology Information (NCBI) BioProject database under accession number PRJNA1391893.

### Exogenous copper supplementation assay

The MIC of NTX against AF293 was assessed in RPMI-1640 supplemented with 0, 10, 50, or 100 μM CuSO_4_ using the broth microdilution method. NTX was two-fold diluted (0.0125–16 µg/mL), inoculated with 1–2.5 × 10^5^ CFU/mL conidia, and incubated at 37°C for 48 h. The MIC was defined as the lowest concentration fully inhibiting visible growth. To quantify copper-dependent modulation of NTX, nine conditions were tested: growth control, medium control, NTX (10 µg/mL), NTX (10 µg/mL) plus 10/50/100 μM CuSO_4_, and CuSO_4_ alone (10/50/100 μM). After 48 h of incubation at 37°C, OD_600_ was measured (BioTek Synergy H1). Relative growth (%) was calculated as: (OD sample − OD blank control) / (OD growth control − OD blank control) × 100%.

### Intracellular ROS detection

Intracellular ROS levels were assessed using a Reactive Oxygen Species Assay Kit (Beyotime, S0033S) following a published protocol with minor modifications (23). Fresh AF293 conidia (1 × 10^7^ CFU/mL) were washed with PBS and incubated with 10 μM DCFH-DA at 37°C in the dark for 30 min. After removing excess probe, conidia were divided into the following groups and incubated for another 30 min at 37°C: blank control (CON), ROSup (positive control), VRC (10 µg/mL), NTX (10 µg/mL), CuSO_4_ (10, 50, 100 μM), and NTX + CuSO_4_. Samples were washed with PBS and transferred to Lysing Matrix B tubes (MP Biomedicals, #6,911,050) for homogenization at 4°C (60 Hz, 30 s × 3 cycles, 30 s interval; JXFSTPRP-CLN). Lysates were centrifuged at 12,000 × *g* for 10 min at 4°C, and supernatants were used to measure fluorescence (Ex 485 nm / Em 528 nm). Relative fluorescence intensity (RFI) was obtained by subtracting background and normalizing to the blank control to reflect ROS changes.

### Statistical analysis

Data are presented as mean ± SD. Statistical analyses and graphing were performed using GraphPad Prism 9, and figures were finalized in Adobe Illustrator. Group differences were evaluated by one-way ANOVA with Tukey’s post hoc test. Survival data were analyzed using the Log-rank (Mantel-Cox) test. RNA-seq differential expression and enrichment analyses were adjusted for multiple testing, and *q* < 0.05 was considered significant. Unless otherwise stated, *P* < 0.05 was regarded as statistically significant. Significance levels were denoted as: *P < 0.05, **P < 0.01, ***P < 0.001, ****P < 0.0001, and ns (not significant).

## RESULTS

### Nitroxoline exhibits antifungal activity against both clinical isolates and AF293 of *A. fumigatus in vitro*

**Fig 2:**
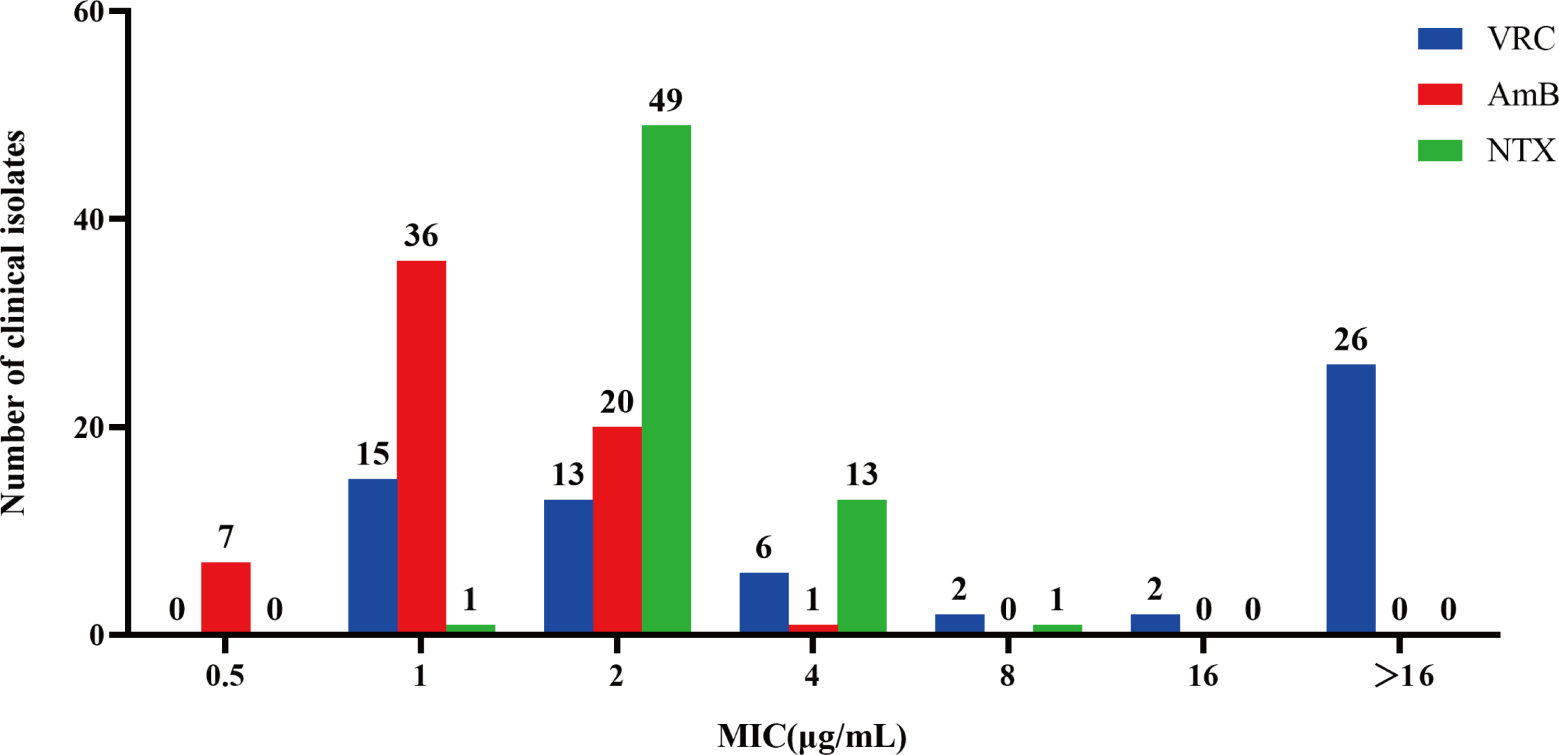
*In vitro* antifungal activity of VRC, AmB, and NTX against *A. fumigatus.* MIC distribution of VRC, AmB, and NTX against 64 clinical isolates.

To evaluate the potential of NTX against *A. fumigatus*, we first determined its antifungal activity against 64 clinical isolates and compared it with the first-line drug VRC and the second-line drug AmB *in vitro*. As shown in Fig. 2, a high rate of resistance to VRC was observed, with 26 out of 64 isolates (40.6%) exhibiting MIC values ≥16 µg/mL. The MICs of AmB were primarily distributed between 1 and 2 µg/mL. In contrast, NTX demonstrated uniform and potent activity against the vast majority of tested strains (49/64, 76.6%), with an MIC of 2 µg/mL. These results indicate that NTX possesses significant antifungal potential against clinical isolates of *A. fumigatus in vitro*, including those resistant to VRC. The specific MICs of VRC, AmB, and NTX for the strain AF293 and the clinical isolates are presented in Table S1. The MICs of the three drugs for AF293 were 2 µg/mL, 1 µg/mL, and 2 µg/mL, respectively. It is important to note that, according to EUCAST guidelines, the VRC MIC of 2 µg/mL for the wild-type strain AF293 may fall at the upper limit of susceptibility within the area of technical uncertainty (ATU) and does not directly equate to a resistant phenotype (24). Furthermore, there are currently no established clinical breakpoints or epidemiological cutoff values (ECOFFs) for NTX against *A. fumigatus*; thus, its MIC is reported here solely to describe *in vitro* inhibitory activity. Given that VRC is the first-line therapy and shares the same MIC as NTX against AF293 under the present testing conditions, these two agents were selected for further comparison.

**Fig 3:**
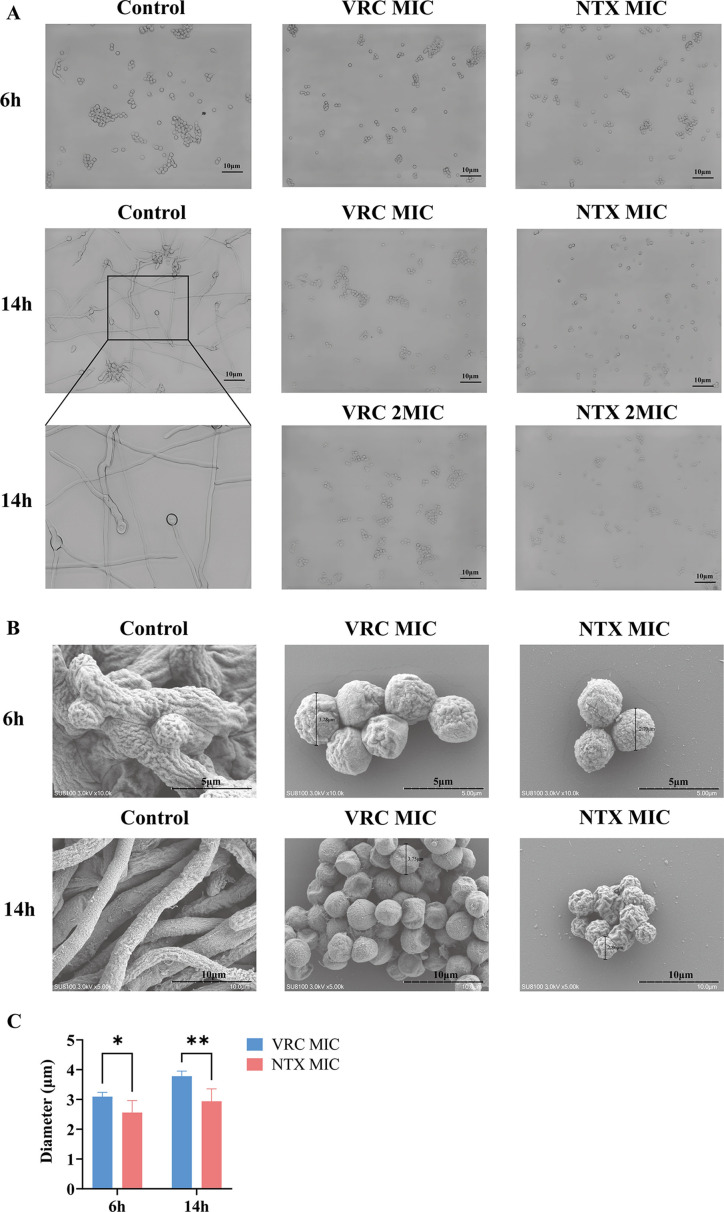
Effects of NTX and VRC on the morphological development of *A. fumigatus*. (**A**) Bright-field images of AF293 under different treatment conditions after 6 h and 14 h of incubation. Control represents the untreated group, while VRC and NTX groups correspond to their 1 × MIC and 2 × MIC treatments. Scale bar = 10 µm. (**B**) SEM images of AF293 under various treatment conditions. Scale bar = 5 µm and 10 μm. (**C**) Statistical analysis of spore diameters measured from SEM images. *P < 0.05, **P < 0.01.

The effects of VRC and NTX on the early developmental stages of AF293 conidia were examined by microscopy (Fig. 3A). After 6 h of cultivation, the control group showed extensive conidial swelling, adhesion, and aggregation, with a few conidia having already germinated. In contrast, after 6 h of exposure to VRC and NTX at their respective MICs, conidial adhesion and aggregation were markedly reduced, and no germinated conidia were observed. After 14 h, the control conidia had germinated and formed abundant hyphae. In contrast, no germination or hyphal formation was observed in the VRC- and NTX-treated groups, where conidia remained in a state of adhesion, aggregation, or as individual conidia. These morphological changes indicate that NTX significantly inhibits the germination of AF293 conidia into hyphae. High-resolution SEM further characterized conidial morphology (Fig. 3B). After 6 h of incubation, conidia in the control group exhibited pronounced swelling and the emergence of germination protrusions, with some already extending short hyphae. In contrast, conidia in both the VRC group and NTX group retained a spherical shape, showing no signs of germination. After 14 h, the Control group developed abundant branched hyphae that formed a dense mycelial network, whereas conidia in the VRC group and NTX group remained ungerminated, displaying only limited aggregation and adhesion. Notably, conidia in the NTX group exhibited a visibly shrunken surface after 14 h, in contrast to those in the VRC group. Quantitative analysis of conidial diameters based on SEM images (Fig. 3C) revealed that after 6 h of treatment, the mean diameters of conidia in the VRC group and NTX group were 3.09 ± 0.15 µm and 2.56 ± 0.40 µm, respectively, showing a statistically significant difference (*P* < 0.05). After 14 h, the diameters increased to 3.78 ± 0.17 µm and 2.94 ± 0.42 µm, respectively, with the difference becoming more pronounced (*P* < 0.01). Compared with VRC, NTX markedly delayed conidial swelling, indicating a stronger inhibitory effect on germination and early growth of AF293 conidia.

### Nitroxoline exhibits therapeutic efficacy in the IPA mouse model *in vivo*

**Fig 4:**
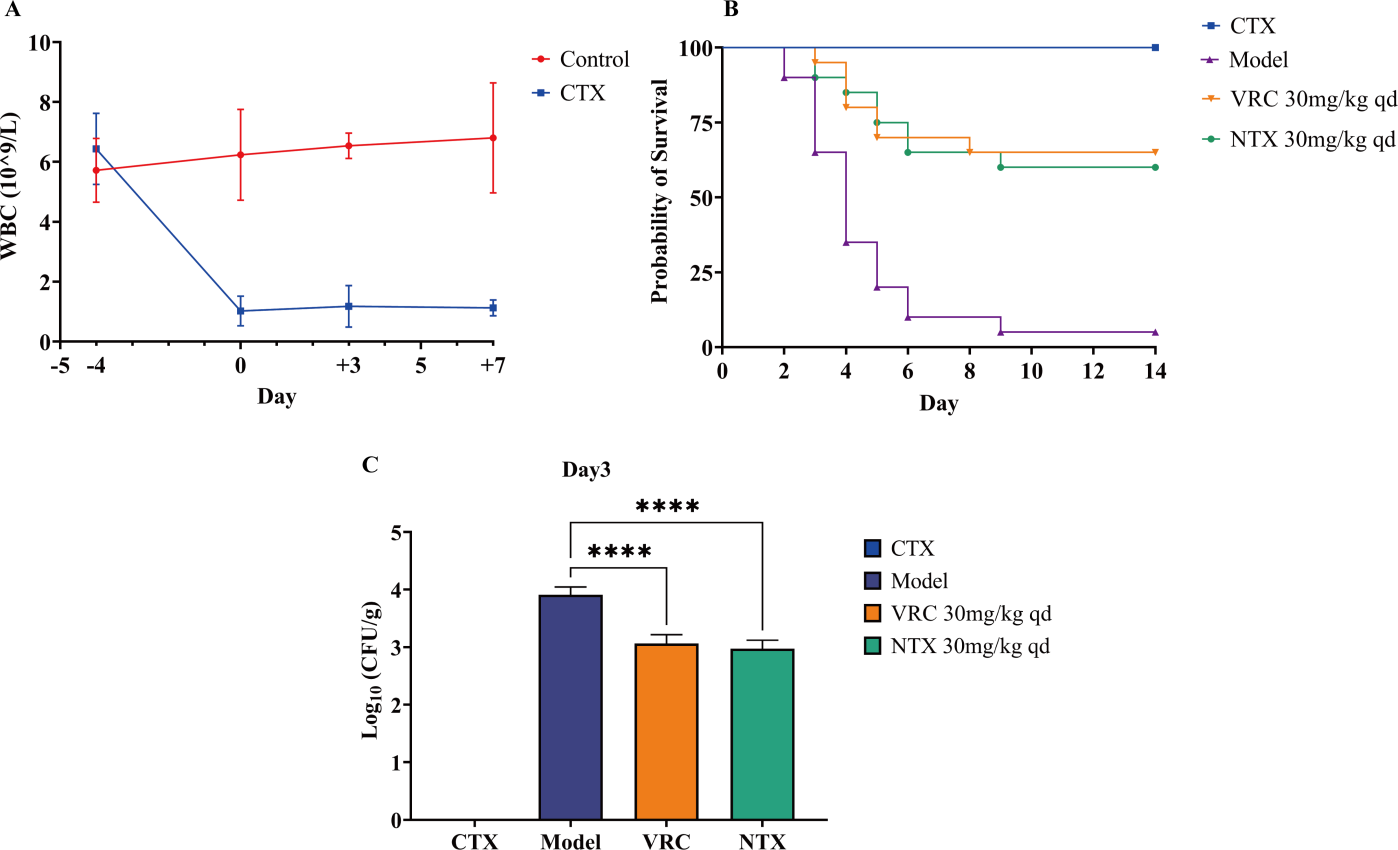
*In vivo* therapeutic efficacy of NTX in IPA mouse model. (**A**) Dynamic changes in peripheral WBC counts in immunosuppressed and immunocompetent control mouse. (**B**) Fourteen-day survival curves of mouse in different groups: CTX group, Model group, VRC group, and NTX group. (**C**) Fungal burden in lung tissues on day 3 post-infection. ****P < 0.0001.

The *in vivo* antifungal efficacy of NTX was assessed using an established immunosuppressed mouse model, with immunosuppression first verified by monitoring peripheral WBC counts (Fig. 4A). Blood samples were collected from the orbital vein on days −4, 0, +3, and +7 to assess the level of immunosuppression during the experimental period. The results showed that WBC counts in the CTX group remained persistently and significantly lower than those in the immunocompetent control group (*P* < 0.0001), confirming that a stable and sustained immunosuppressive state was successfully induced for the subsequent IPA infection and treatment experiments. The survival outcomes of the different treatment groups were then assessed over 14 days (Fig. 4B). Mice subjected to CTX-induced immunosuppression without infection exhibited a 100% survival rate, confirming that the immunosuppressive regimen itself was not lethal. In contrast, the Model group experienced a rapid decline in survival, with only a 5% survival rate by day 14, indicating successful infection. Both VRC (30 mg/kg/day) and NTX (30 mg/kg/day) treatments significantly improved the survival of infected mice, with 14-day survival rates increasing to 65% (*P* < 0.0001) and 60% (*P* < 0.0001), respectively.

**Fig 5:**
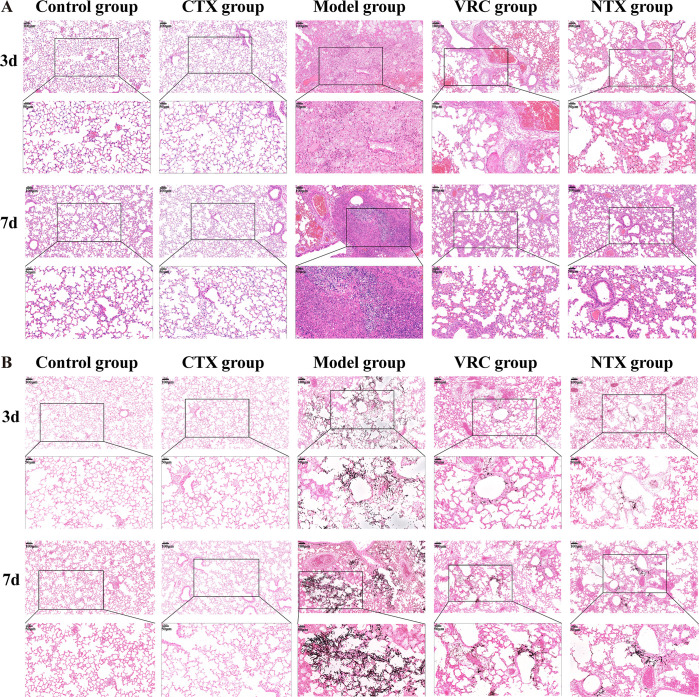
Histopathological analysis of lung tissues from mice with IPA on days 3 and 7 post-infection. (**A**) Representative H&E-stained images of lung tissues from the Control, CTX, Model, VRC, and NTX groups on days 3 and 7 post-infection. Scale bar = 100 µm. Lower panels show higher-magnification views of the boxed regions, scale bar = 50 µm. (**B**) Representative PASM-stained lung sections from days 3 and 7, illustrating hyphal invasion in each group. Scale bars are 100 µm for the overview images and 50 µm for the higher-magnification views of the boxed areas.

To further investigate the antifungal effects of the treatments, fungal burdens in lung tissues were quantified. On day 3 post-infection (Fig. 4C), both VRC group and NTX group showed a significant reduction in fungal burden compared with the Model untreated control group (*P* < 0.0001). Lung histopathology was evaluated by H&E and PASM staining (Fig. 5). H&E staining (Fig. 5A) showed that the pulmonary lobular structure of mice in the Control and CTX groups was well preserved, with clear alveolar spaces and no obvious exudation, inflammatory cell infiltration, or interstitial edema. In contrast, lung tissues from the Model group on days 3 and 7 post-infection exhibited marked inflammatory responses, including infiltration of inflammatory cells, thickening of alveolar walls, pulmonary hemorrhage, and interstitial edema, confirming the successful establishment of the IPA model. Notably, lung structures in the VRC group and NTX group were relatively intact, with clearly defined bronchiolar walls and substantially reduced inflammatory responses compared with the Model group. PASM staining (Fig. 5B) revealed no spores or hyphae in the lungs of Control and CTX groups. By day 3 post-infection, most spores in the Model group had germinated into hyphae, with only a few remaining as ungerminated spores. In contrast, the majority of spores in the VRC group and NTX group remained ungerminated, with minimal hyphal development. By day 7 post-infection, extensive hyphal invasion was observed in the Model group, whereas only localized hyphal presence was detected in the lungs of the VRC group and NTX group, indicating that NTX effectively inhibited *A. fumigatus* invasion and spread *in vivo*.

### Mechanism of action of NTX against *A. fumigatus*

**Fig 6:**
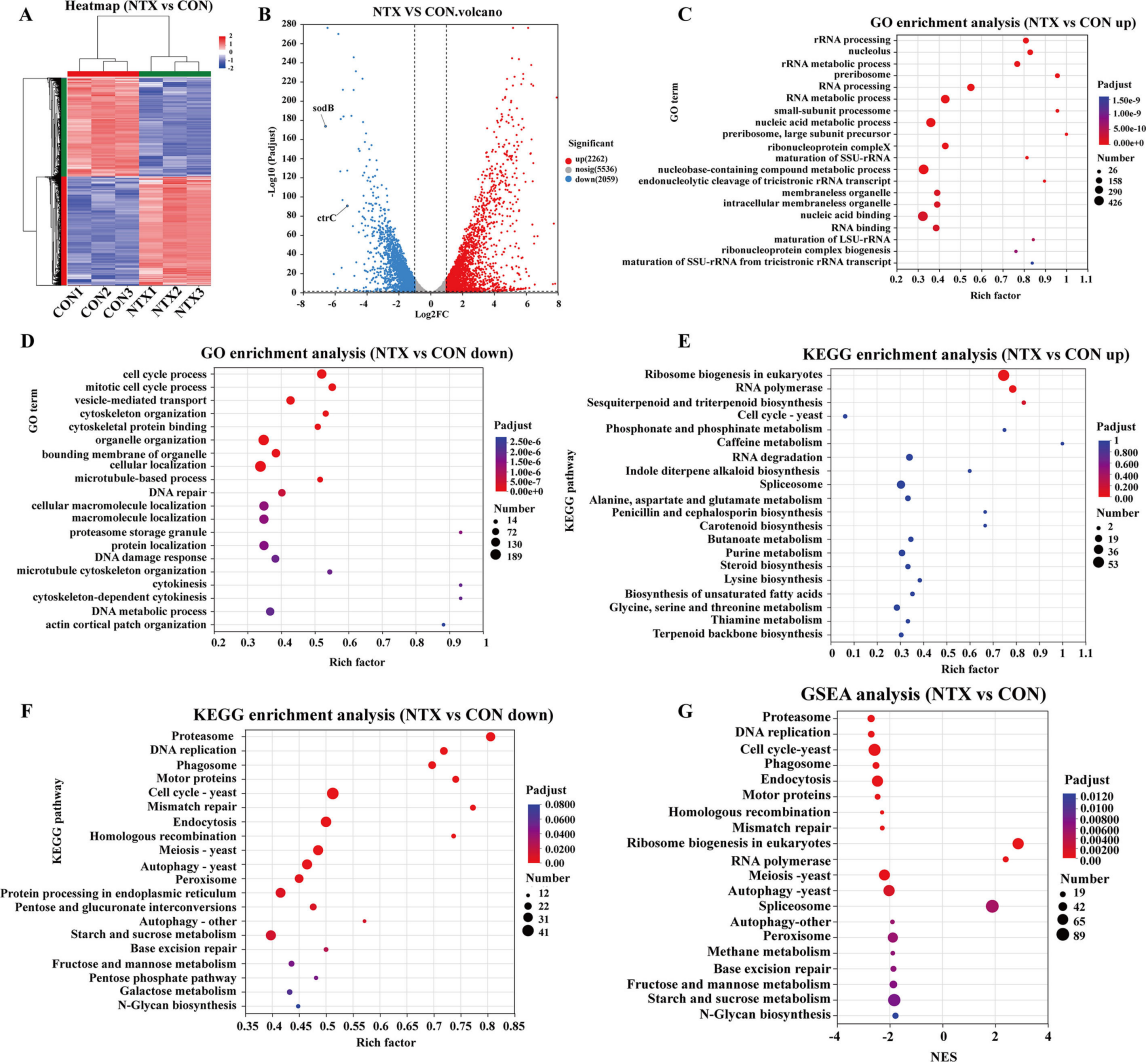
Transcriptomic profiling of *A. fumigatus* conidia treated with NTX. (**A**) Heatmap of DEGs between the NTX and CON groups (*n* = 3). Red and blue indicate upregulated and downregulated genes, respectively. (**B**) Volcano plot of DEGs, with significantly upregulated and downregulated genes highlighted in red and blue. Key genes *sodB* and *ctrC* are labeled. (**C, D**) GO enrichment analysis of upregulated (**C**) and downregulated (**D**) DEGs. (**E, F**) KEGG pathway enrichment analysis of upregulated (**E**) and downregulated (**F**) DEGs. (**G**) GSEA showing significant enrichment of gene sets related to the proteasome, DNA replication, cell cycle, and peroxisome.

RNA-seq was performed on conidia with or without NTX to investigate its antifungal mechanism. NTX treatment caused extensive transcriptional remodeling (Fig. 6A and B), with particularly notable downregulation of the oxidative stress-related gene *sodB* (*sod3*, *AFUA_1G14550*) and the copper transporter gene *ctrC* (*CTR3*, *AFUA_2G03730*). GO enrichment showed that upregulated genes were primarily associated with rRNA processing (Fig. 6C), whereas downregulated genes were enriched in cell cycle-related processes (Fig. 6D). KEGG analysis revealed activation of ribosome biogenesis and suppression of proteasome, DNA replication, cell cycle, and peroxisome pathways (Fig. 6E and F). GSEA further confirmed these pathway alterations (Fig. 6G and 1). Overall, these transcriptomic results suggest that NTX may exert antifungal activity by disrupting protein homeostasis, impairing cell-cycle progression, and perturbing fungal oxidative-stress balance.

**Fig 7:**
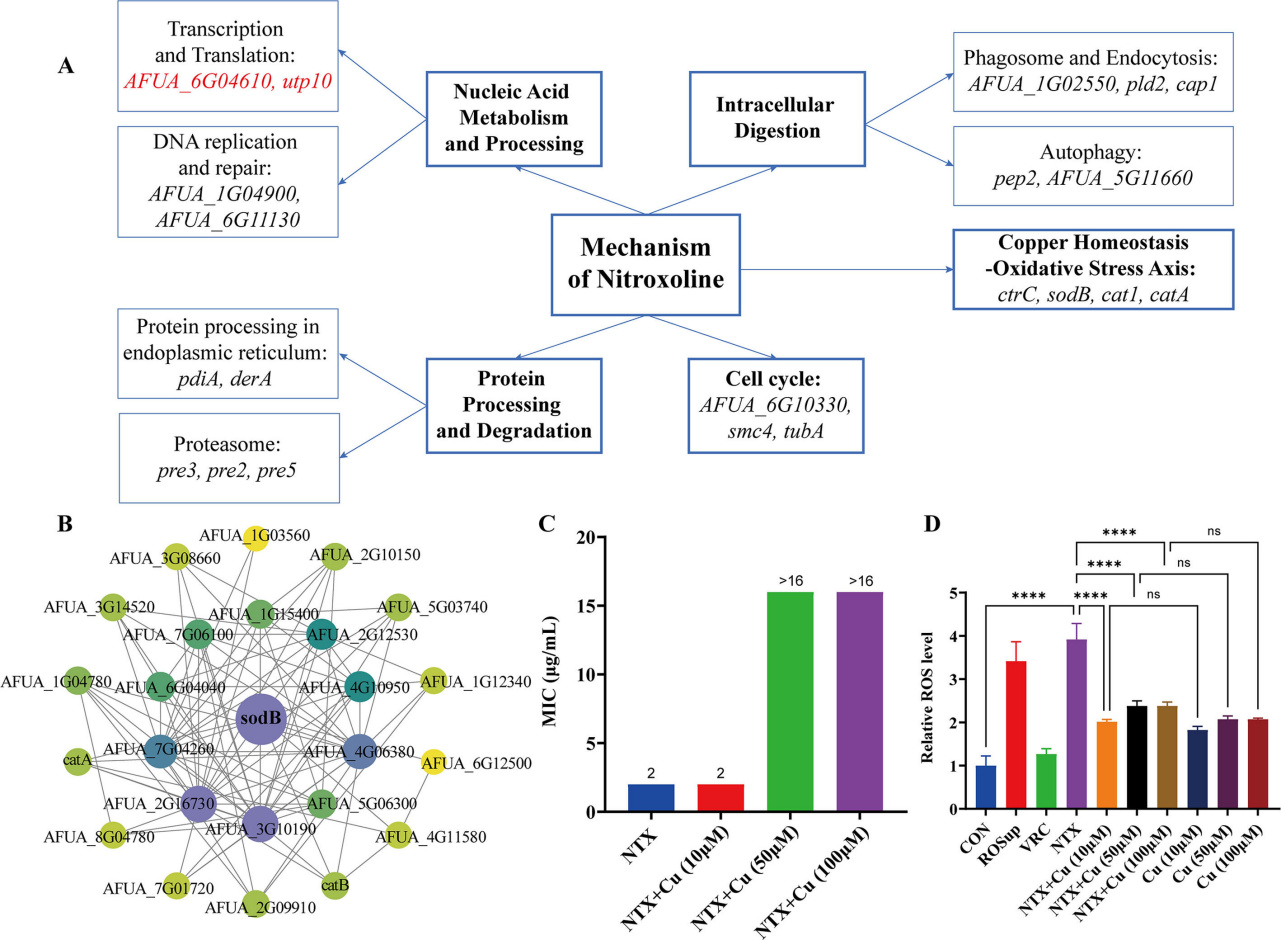
Investigation into the mechanism of action of NTX. (**A**) Schematic summary of NTX-affected functional modules. Red and black indicate upregulated and downregulated genes within each module. (**B**) PPI network of significantly downregulated genes in the peroxisome pathway. (**C**) MIC of NTX against *A. fumigatus* under varying Cu concentrations. (**D**) Relative ROS levels across treatment groups. ****P < 0.0001; ns, not significant.

We then integrated the transcriptional data to construct a functional map highlighting key biological modules perturbed by NTX (Fig. 7A). The analysis revealed that NTX perturbed several key biological processes, including protein processing and degradation, intracellular digestion, cell cycle regulation, nucleic acid metabolism and processing, and a central regulatory axis concerning copper homeostasis and oxidative stress. Notably, the expression of both the copper transporter *ctrC* and the superoxide dismutase *sodB* within this axis was significantly downregulated. To delve deeper into the functional relationships within the oxidative stress pathway, we constructed a PPI network for the proteins encoded by significantly downregulated genes in the peroxisome pathway (Fig. 7B). Analysis positioned SodB as a central regulatory hub within this network, while the coordinated suppression of *ctrC* and *sodB* indicates that NTX perturbs a copper homeostasis-linked redox regulatory axis.

The potential role of copper homeostasis in NTX activity was further examined by assessing its antifungal effect in the presence of exogenous copper ions. MIC determination demonstrated that the addition of 10 µM, 50 µM, or 100 µM copper elevated the MIC of NTX against AF293 from 2 µg/mL to greater than 16 µg/mL, indicating that exogenous copper significantly antagonized the inhibitory effect of NTX (Fig. 7C). For quantitative assessment of this copper-rescuing effect, we measured OD_600_ and calculated the relative growth rate under different treatment conditions (Table 1). The results showed that NTX (10 µg/mL) alone completely inhibited fungal growth, whereas the addition of exogenous copper led to a concentration-dependent restoration of growth. The relative growth rate was merely 3% with 10 µM Cu, but recovered to 74% and 103% with 50 µM and 100 µM Cu, respectively. In contrast, copper supplementation alone (10–100 µM) showed no significant effect on fungal growth (relative growth rate 91–98%). These results confirm that copper ions can significantly reverse the antifungal effect of NTX, underscoring the crucial role of disrupted intracellular copper homeostasis in the mechanism of action of NTX. Subsequently, detection of intracellular ROS levels revealed a significant increase in the NTX alone group compared to the control (*P* < 0.0001) (Fig. 7D). This NTX-induced ROS accumulation was significantly mitigated by exogenous copper compensation (*P* < 0.0001). Collectively, these findings support a mechanistic framework in which NTX perturbs copper homeostasis, thereby compromising ROS detoxification capacity, provoking excessive ROS accumulation, and ultimately leading to fungal cell death.

**TABLE 1:** Effects of nitroxoline and exogenous copper on the growth of *A. fumigatus*

Group	OD_600_ (Mean ± SD)	Relative growth (%)
Control positive	1.00 ± 0.09	100
Control negative	0	0
NTX (10 µg/mL)	0.00 ± 0.1	0
NTX (10 µg/mL) + Cu (10 µM)	0.03 ± 0.01	3
NTX (10 µg/mL) + Cu (50 µM)	0.74 ± 0.01	74
NTX (10 µg/mL) + Cu (100 µM)	1.03 ± 0.03	103
Cu (10 µM)	0.98 ± 0.06	98
Cu (50 µM)	0.96 ± 0.03	96
Cu (100 µM)	0.91 ± 0.02	91

Finally, phylogenetic comparisons and molecular docking analyses were performed to explore potential interactions between NTX and the key proteins CtrC and SodB (Fig. S2). Phylogenetic trees demonstrated high conservation of both CtrC and SodB among closely related fungi, including *Aspergillus* and *Penicillium* species (Fig. S2A and B). Molecular docking predicted that NTX could form stable conformations within the functional pockets of both CtrC and SodB. However, the calculated binding affinities were relatively modest (approximately −4.7 kcal/mol) (Fig. S2C and D). This computational analysis suggests that NTX likely does not function through high-affinity binding to a single protein target but rather achieves its antifungal effect by perturbing the broader copper homeostasis and oxidative stress balance.

## DISCUSSION

IPA remains a major clinical challenge due to its high mortality, highlighting the urgent need for agents with novel mechanisms (2). Here, we show that the approved antibiotic NTX exhibits potent antifungal activity against *A. fumigatus*, including VRC-resistant clinical isolates, with MICs comparable to or lower than those of VRC (Fig. 2). In a mouse IPA model, NTX significantly improved survival and reduced pulmonary fungal burdens (Fig. 4 and 5), supporting its potential repurposing as an effective anti-*A. fumigatus* agent. Mechanistically, NTX disrupts intracellular copper homeostasis, leading to oxidative stress, which underlies its unique antifungal efficacy. These results not only validate NTX’s pharmacological potential but also suggest metal homeostasis as a promising target for antifungal therapy.

The findings of this study reveal a dialectical relationship with previous reports on the antimicrobial mechanisms of 8-HQ derivatives, demonstrating both conceptual concordance and mechanistic divergence, thereby highlighting the distinctive mode of action of NTX. Consistent with the observations of Festa et al. in *Cryptococcus neoformans*, our results confirm the pivotal role of the 8-HQ in mediating metal-dependent antimicrobial activity (25). For *A. fumigatus*, although a preliminary report of *in vitro* activity against seven clinical strains was made by Simon et al., our investigation provides a substantial extension by including a significantly larger collection of clinical strains (64 versus 7), coupled with *in vivo* and mechanistic investigations, offering considerably more conclusive evidence (11). Similar to other 8-HQ derivatives, NTX is capable of forming metal complexes with Cu^2+^ ions, subsequently promoting ROS generation and triggering oxidative stress responses (25, 26). Moreover, the antitumor properties of NTX have been attributed to the nitro substituent, which generates nitrogen-centered radicals and perturbs redox balance, further elevating intracellular ROS levels (25, 27). Together, these observations support the notion that metal chelation and ionophoric behavior constitute a unifying pharmacological basis underlying the biological activities of this chemical class. In contrast, our findings in the *A. fumigatus* model revealed a critical mechanistic distinction: NTX’s antifungal activity does not rely primarily on copper complexation-mediated toxicity. Previous studies have shown that exogenous Cu^2+^ often potentiates the antimicrobial effect of 8-HQ derivatives by facilitating intracellular copper overload and subsequent microbial killing (15, 25, 28). Unexpectedly, we observed that copper supplementation markedly attenuated the antifungal efficacy of NTX (Table 1, Fig. 7C). This finding suggests that NTX may instead act through a chelation-deprivation mechanism, reducing bioavailable copper within fungal cells and thereby disrupting antioxidant defense systems (29). This mechanism aligns closely with the competitive chelation phenomena reported by Pelletier et al. in bacterial models, where Mg^2+^ and Mn^2+^ ions diminished NTX activity through site competition (30). Similarly, Cherdtrakulkiat et al. observed that the presence of Cu²^+^ significantly increased the MICs of Enterobacteriaceae, further indicating that metal ions may function as essential cofactors supporting microbial survival (29). Collectively, these results provide cross-species experimental support for the metal sequestration or metal deprivation model and uncover a mechanistic connection between NTX activity and fungal metal homeostasis. Importantly, this mechanism complements rather than contradicts the classical metal-loading toxicity paradigm, while extending our understanding of the metal dependence of 8-HQ derivatives across diverse microbial systems (15, 31, 32). Taken together, these findings indicate a context-dependent, biphasic relationship between NTX and metal ions: while chelation of essential metal cofactors is necessary for antimicrobial action, competitive metal binding can neutralize NTX activity instead of promoting metal-mediated toxicity, highlighting a complex drug-metal-pathogen interplay (12, 15, 29, 33, 34).

By integrating transcriptomic profiling, copper antagonism assays, and biochemical validation, we delineate a mechanistic framework in which NTX exerts antifungal activity against *A. fumigatus* through a central copper homeostasis disruption-oxidative stress axis (15, 25). Acting as a potent metal chelator, NTX displays antimicrobial potency that is almost entirely dependent on its chelating capability (12, 35). Transcriptomic analysis showed that pathways related to proteasome and cell cycle were reflective of the fungus’s overall physiological decline and downstream phenotypic outcomes, such as cell death, under drug exposure (Fig. 6F). In contrast, the robust activation of the peroxisome pathway was particularly informative, pointing toward a systemic perturbation of the cellular redox steady state that constitutes the initial stress signal (Fig. 6F). Notably, the downregulation of *ctrC*, encoding a key copper transporter, and *sodB*, encoding the gene for superoxide dismutase (Fig. 6B), emerged as a molecular signature of disrupted metal homeostasis, strongly implicating copper deprivation as a trigger of oxidative stress. This finding aligns with prior studies demonstrating that 8-HQ derivatives can sequester metal ions, restrict their bioavailability, and inhibit metalloenzymes such as superoxide dismutase (SOD)(27, 29). Copper supplementation experiments conclusively validated the functional metal deprivation model: the introduction of exogenous Cu^2+^ partially mitigated the drug’s effects by restoring fungal growth and normalizing ROS levels, thereby confirming that NTX acts via chelation-mediated depletion of essential copper rather than through the induction of a metal-overload state (Table 1, Fig. 7D). It is important to note that our ROS quantification was primarily conducted at the conidial stage. This decision was based on the higher technical reproducibility of conidial assays for capturing early drug-induced stress responses, as well as our morphological observation that NTX significantly inhibits conidial germination (Fig. 3). While ROS levels in conidia may not fully reflect the physiological status of mature hyphae during invasive infection, these data effectively highlight the oxidative damage triggered by NTX during the critical initial stages of fungal development. Extrapolating from previous studies demonstrating that NTX competitively coordinates the metal-binding site of bacterial methionine aminopeptidase (MetAP) to inhibit growth, we propose a dual-action competitive metal-binding mechanism for NTX in *A. fumigatus* (29, 36). Specifically, it forms non-physiological complexes with copper ions, thus reducing their bioavailability, while simultaneously hindering the acquisition of essential cofactors by copper-dependent antioxidant systems (29, 36). This combined interference precipitates oxidative stress and subsequent cellular damage. Therefore, the integrated evidence from transcriptomics, phenotypic rescue, and oxidative stress assays suggests that perturbation of metal homeostasis, particularly within the copper-associated redox balance, contributes to the antifungal activity of NTX. However, given the multimetal-binding property of NTX as an 8-hydroxyquinoline (8-HQ) derivative, the present data do not exclude alternative or complementary mechanisms. Thus, we propose the “copper homeostasis-oxidative stress axis” as a biologically plausible model that is consistent with our observations, rather than a definitive or exclusive mechanistic conclusion. This model broadens the pharmacological understanding of 8-HQ derivatives in the antifungal domain and provides a rationale for developing novel antifungal agents that target metal acquisition and defense pathways (15, 36).

The central merit of this investigation lies in systematically elucidating the antifungal mechanism of NTX against *A. fumigatus* through the novel perspective of metal homeostasis imbalance. By integrating the results, we established a multi-layered and mutually corroborating evidence chain. This successfully reveals the unique mode of action—functional copper starvation—induced by NTX in *A. fumigatus*. This approach fundamentally differs from traditional research frameworks focused on direct targeting of the cell wall, cell membrane, or nucleic acid synthesis, thereby providing a robust theoretical and conceptual basis for designing novel anti-*A. fumigatus* strategies that leverage metal homeostasis regulation (37). Furthermore, our multi-dimensional validation system, which combines molecular transcript analysis, computational modeling, oxidative stress functional assays, and phenotypic analysis, significantly enhances the credibility and persuasiveness of these conclusions. However, several outstanding questions remain that will be the focus of our continued investigation. Firstly, while the transcriptomic results showing the downregulation of *CtrC* and *SodB* support the metal deprivation hypothesis, definitive causal verification through genetic manipulation is currently lacking. Concurrently, changes in metal ion dynamics are primarily inferred from functional assays, necessitating direct quantitative evidence at the cellular or subcellular level. To address these critical mechanistic gaps, our forthcoming research program will focus on utilizing combined metal tracing and metabolomic technologies to finely map the NTX-induced copper dynamic distribution and the cellular response network; simultaneously, we will employ genetically modified strains and host models to dissect the precise causal chain between copper homeostasis disruption and oxidative stress. Secondly, although the *in vivo* infection model validated the anti-infective efficacy of NTX, further pharmacological and translational characterization is warranted. While our evaluation relied on classical CFU quantification and histopathology—an established approach providing consistent evidence of efficacy—incorporating qPCR-based DNA quantification and systematic pharmacokinetic (PK) analysis, including tissue distribution in relevant target organs, would allow for a more precise assessment of drug exposure and PK/PD relationships (38). Moreover, while the lack of apparent acute toxicity in this study, combined with the established clinical safety profile of NTX, provides a preliminary foundation for its translational potential, comprehensive preclinical toxicological evaluations remain a necessary requirement for future studies (8). Notably, NTX exhibited relatively consistent inhibitory activity across clinical isolates with varying levels of voriconazole susceptibility, with MIC values predominantly ranging from 2 to 4 µg/mL (Fig. 2). Given that its antifungal activity is likely mediated through disruption of metal homeostasis rather than classical ergosterol biosynthesis or cell wall synthesis pathways, this distinct mode of action suggests that NTX may have the potential to overcome certain forms of resistance to currently available antifungal agents. Accordingly, future studies will prioritize the systematic evaluation of NTX efficacy in genetically well-characterized azole- or echinocandin-resistant *A. fumigatus* strains. In addition, from a host-pathogen interaction perspective, we will further investigate whether NTX can synergistically enhance antifungal efficacy in complex *in vivo* settings by modulating host-mediated nutritional immunity, such as metal chelation and sequestration processes. As these investigations progress, we anticipate further completing the mechanistic blueprint of NTX, thereby laying a more solid theoretical and translational foundation for developing next-generation antifungals that target metal homeostasis (15, 36).

In summary, this study demonstrates that NTX has promising activity against *A. fumigatus in vitro* and *in vivo* and disrupts metal homeostasis and induces oxidative stress in *A. fumigatus*, highlighting the copper-oxidative stress axis as a key vulnerability. These findings redefine the antifungal potential of 8-hydroxyquinoline derivatives and provide mechanistic support for overcoming antifungal drug resistance. Overall, our work positions NTX as a promising repurposed agent for treating *A. fumigatus* infections and underscores metal homeostasis modulation as a novel strategy for developing next-generation antifungal therapeutics with translational relevance.

## Data Availability

The data that support the findings of this study are available from the corresponding author upon reasonable request.

## SUPPLEMENTAL MATERIAL

Supplemental material aac.01831-25-s0001.docx Table S1; Fig. S1 and S2.

ASM does not own the copyrights to Supplemental Material that may be linked to, or accessed through, an article. The authors have granted ASM a non-exclusive, world-wide license to publish the Supplemental Material files. Please contact the corresponding author directly for reuse.
