# Supplementary material for: Nitroxoline exerts potent anti-Aspergillus fumigatus activity by disrupting copper homeostasis and inducing oxidative stress
Source: Antimicrob Agents Chemother. 2026 Mar 31;70(5):e01831-25. doi: 10.1128/aac.01831-25 (PMC13148046; doi:10.1128/aac.01831-25)
Supplement: Supplemental material — Table S1; Fig. S1 and S2. [file aac.01831-25-s0001.docx]

**Supporting Table**

**Table S1. MIC of VRC, AmB, and NTX against AF293 and Clinical Isolates of *A. fumigatus*.**

| Isolate ID | VRC (μg/mL) | AmB (μg/mL) | NTX (μg/mL) |
| --- | --- | --- | --- |
| AF293 | 2 | 1 | 2 |
| P03 | > 16 | 1 | 2 |
| P04 | 4 | 1 | 2 |
| P05 | > 16 | 0.5 | 2 |
| P06 | 1 | 1 | 2 |
| P07 | 2 | 0.5 | 2 |
| P09 | 1 | 1 | 2 |
| P10 | 1 | 1 | 2 |
| P12 | 16 | 1 | 2 |
| P14 | 2 | 2 | 2 |
| P15 | 1 | 1 | 2 |
| P16 | 2 | 1 | 2 |
| P17 | 1 | 2 | 4 |
| P18 | 1 | 1 | 2 |
| P19 | 1 | 1 | 2 |
| P20 | 1 | 1 | 2 |
| P21 | 4 | 1 | 4 |
| P22 | > 16 | 1 | 2 |
| P23 | > 16 | 1 | 2 |
| P24 | 2 | 1 | 2 |
| P26 | 8 | 0.5 | 4 |
| P29 | 2 | 2 | 4 |
| P30 | 2 | 2 | 4 |
| P31 | 4 | 1 | 2 |
| P32 | 16 | 2 | 4 |
| P33 | 1 | 1 | 2 |
| P34 | > 16 | 1 | 2 |
| P35 | > 16 | 2 | 2 |
| P36 | 4 | 0.5 | 2 |
| P37 | > 16 | 2 | 2 |
| P38 | 2 | 1 | 2 |
| P40 | 2 | 2 | 2 |
| P41 | > 16 | 0.5 | 2 |
| P42 | > 16 | 1 | 2 |
| P43 | > 16 | 1 | 2 |
| P44 | 2 | 0.5 | 2 |
| P45 | > 16 | 1 | 4 |
| P46 | > 16 | 2 | 4 |
| P47 | > 16 | 2 | 8 |
| P49 | 1 | 2 | 4 |
| P50 | > 16 | 1 | 2 |
| P51 | 1 | 2 | 4 |
| P53 | 1 | 1 | 2 |
| P54 | 1 | 1 | 2 |
| P55 | > 16 | 1 | 2 |
| P56 | > 16 | 2 | 2 |
| P57 | > 16 | 2 | 2 |
| P58 | 8 | 1 | 2 |
| P59 | 2 | 1 | 2 |
| P61 | > 16 | 0.5 | 2 |
| P62 | 1 | 1 | 2 |
| P64 | > 16 | 1 | 2 |
| P65 | 4 | 1 | 2 |
| P66 | 4 | 1 | 1 |
| P69 | > 16 | 2 | 4 |
| P70 | > 16 | 1 | 4 |
| P71 | > 16 | 2 | 4 |
| P72 | > 16 | 2 | 2 |
| P74 | > 16 | 1 | 2 |
| P75 | > 16 | 1 | 2 |
| P76 | > 16 | 2 | 2 |
| P77 | 1 | 1 | 2 |
| P78 | 2 | 2 | 2 |
| P79 | 2 | 4 | 2 |
| P80 | 2 | 2 | 2 |

Abbreviations: MIC, minimum inhibitory concentration; VRC, voriconazole; AmB, amphotericin B; NTX, nitroxoline.

**Supporting Figures**


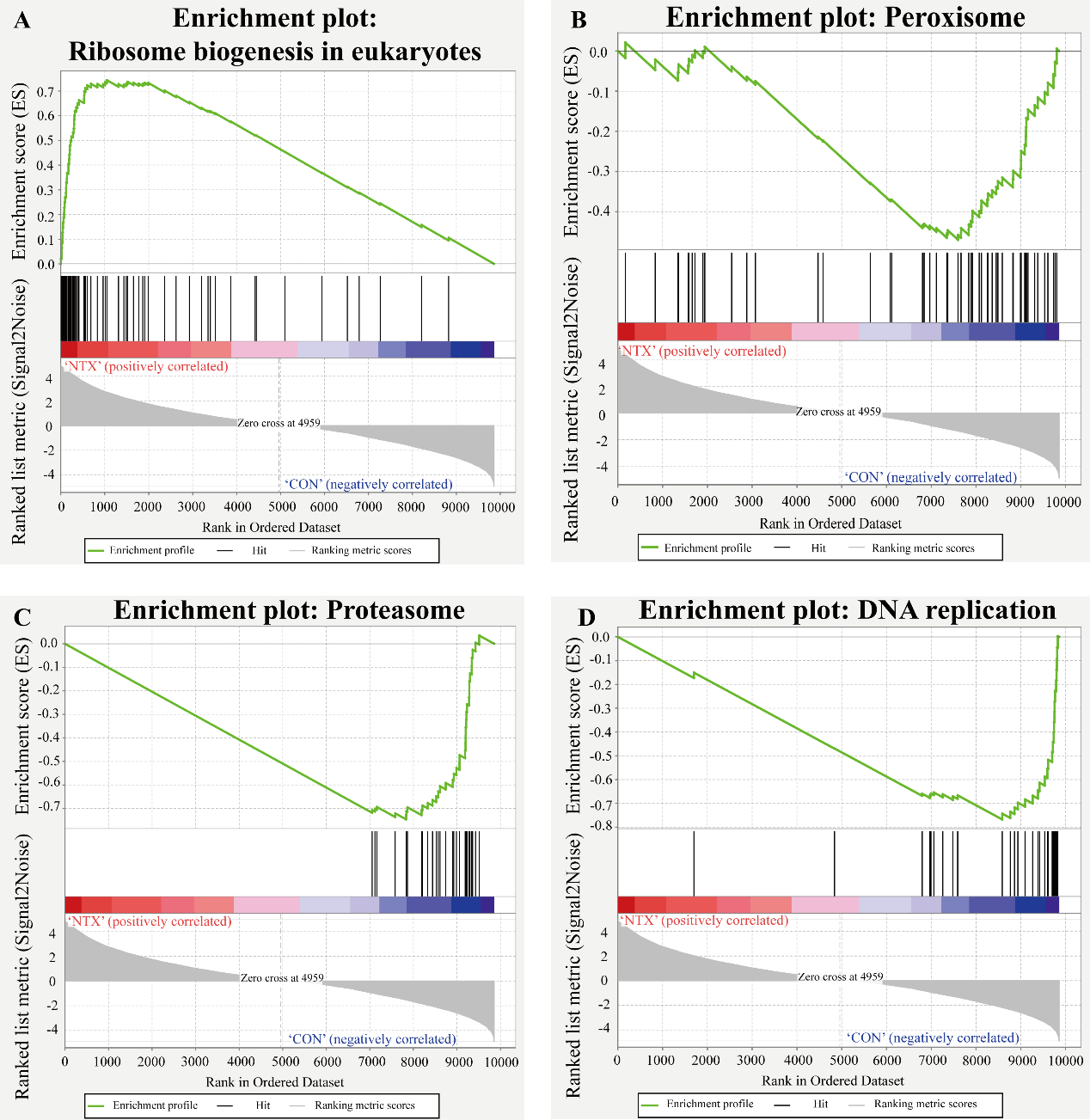


**Figure S1.** GSEA enrichment plots showing significant enrichment in ribosome biogenesis in eukaryotes (A), peroxisome (B), proteasome (C), and DNA replication (D).


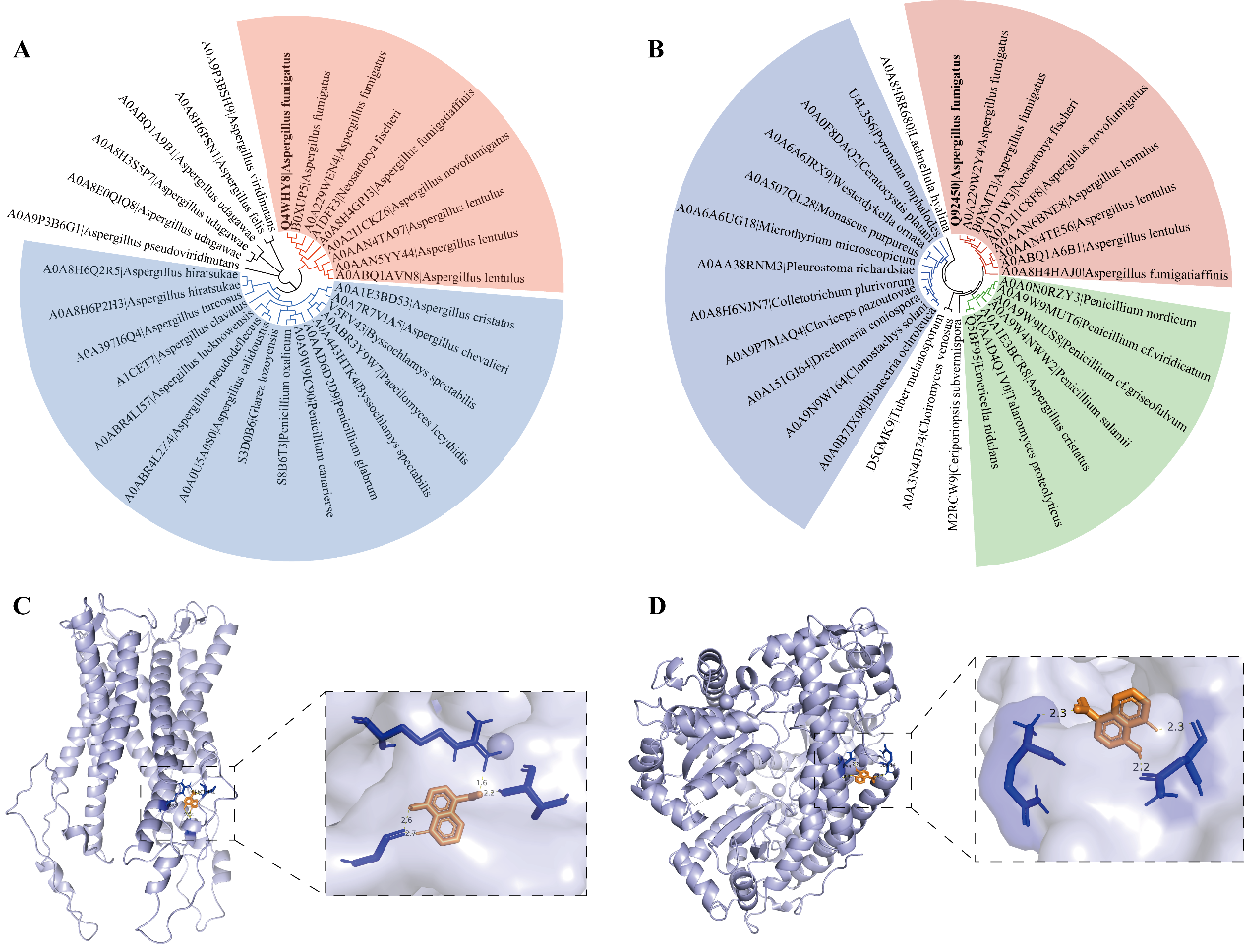


**Figure S2. Phylogenetic and molecular docking analysis of *A. fumigatus* CtrC and SodB proteins.** (A) Phylogenetic tree of the CtrC protein from *A. fumigatus* and its homologs in other *Aspergillus* and *Penicillium* species. (B) Phylogenetic tree of the SodB protein from *A. fumigatus* and its homologs in other fungi, including *Aspergillus*, *Penicillium*, *Microthyrium*, and *Pleurostoma*. (C) Molecular docking model of nitroxoline (orange) bound to the CtrC protein. The overall protein structure (gray) and the binding pocket (yellow) are shown on the left, while the right panel provides a close-up view highlighting the interacting residues and binding distances (in Å). (D) Molecular docking model of nitroxoline (orange) bound to the SodB protein. The overall structure (gray) and the binding pocket (yellow) are displayed on the left, with a detailed view of the binding pose and key interaction distances (in Å) shown on the right.
